# Whose waters, whose nutrients? Knowledge, uncertainty, and controversy over eutrophication in the Mar Menor

**DOI:** 10.1007/s13280-023-01846-z

**Published:** 2023-04-07

**Authors:** Violeta Cabello, Marcela Brugnach

**Affiliations:** Basque Centre for Climate Change, Headquarters Building 1, 1st floor | Scientific Campus of the University of the Basque Country, 48940 Leioa, Biscay Spain

**Keywords:** Ambiguity, Eutrophication, Hydro-social problems, Mar Menor, Narratives, Relational uncertainty

## Abstract

This work explores the role of knowledge claims and uncertainty in the public dispute over the causes and solutions to nonpoint-driven overfertilization of the Mar Menor lagoon (Spain). Drawing on relational uncertainty theory, we combine the analysis of narratives and of uncertainty. Our results show two increasingly polarized narratives that deviate in the causes for nutrient enrichment and the type of solutions seen as effective, all of which relate to contested visions on agricultural sustainability. Several interconnected uncertainties are mobilized to dispute the centrality of agriculture as a driver for eutrophication and to confront strategies that may hamper productivity. Yet, both narratives rest on a logic of dissent that strongly relies on divergent knowledge to provide legitimacy, ultimately reinforcing contestation. Transforming the ongoing polarization dynamics may require different inter- and transdisciplinary approaches that focus on sharing rather than assigning responsibility and that unpack rather than disregard existing uncertainties.

**Supplementary Information:**

The online version contains supplementary material available at 10.1007/s13280-023-01846-z.

## Introduction

Recent insights from social and interdisciplinary science discuss eutrophication as a ‘wicked’ hydro-social issue (Rittel and Webber 1973) characterized by scientific controversies, local struggles, rural-coastal polarization and political stagnation (Thornton et al. 2013; Le Moal et al. 2019; Levain et al. 2020). Not only eutrophication lacks a unified definition, but also the approach, scale and type of interventions continue evolving as new aspects of the problem are revealed and gain recognition as a global environmental concern (Thornton et al. 2013; Le Moal et al. 2019). An important turning point in defining this issue was the shift from point-source pollution, mostly urban sewage as the main cause for nutrient enrichment of water environments, to nonpoint pollution from a variety of natural and anthropogenic sources (Nixon 2009; Whitney 2010). This rendered previous technological solutions, and knowledge, ineffective, and raised questions about social, economic, and cultural roots of land-based aquatic overfertilization. As posed by Levain et al. (2020 p. 1), eutrophication cases often deal with “social complexity, multi-scale dynamics, changing perceptions, path dependency, and power relations forming a wall offering little purchase for transformative action.”

As in most complex issues, eutrophication processes are mediated by the politics of knowledge and uncertainty (Brugnach and Ingram 2012; Levain et al. 2020). The difficulty in assigning clear-cut responsibilities in diffuse nutrient pollution, known as ‘the nonpoint pollution dilemma’ (Whitney 2010), is breeding ground for scientific and political disputes. Mascareño et al. (2018) unravel such controversies in the case of red tides in Chiloe Island. They show how the controversy evolved from the cause for shellfish stranding, whether it was related to a toxic red tide event or to the dumping of dead fishes from salmon farming, to then questioning which were the different possible factors behind the red tide. Explanations provided by public authorities relying on scientific knowledge were strongly contested by the experiential knowledge of fishermen and local workers. The situation rapidly evolved to social mobilizations in open conflict with the central government. There are, however, examples of long-standing disputes around eutrophication settled through the production of new knowledge in a sufficiently legitimate way. In the controversy surrounding macroalgal blooms along the coasts of Brittany, Bourblanc (2019) discusses expert assessments as framing exercises that serve to provide a more consensual problem definition. This is illustrated by the creation of new scientific indicators that balanced ecological and economic dimensions, releasing the emphasis on agricultural productivity as a key driver for eutrophication. This movement helped settle a lengthy scientific controversy over which nutrient—nitrogen or phosphorus—was the limiting factor for the proliferation of algae blooms, grounded in divergent knowledge claims.

In fact, knowledge is often mobilized to bear legitimacy in environmental controversies, especially under conditions of uncertainty (Sarewitz 2004; Brugnach et al. 2011). Yet the issue of uncertainty in nonpoint pollution dilemmas is still underexplored. The difficulty in estimating nutrient leakage from distributed origins adds up to epistemic uncertainties in biogeochemical modeling along the land-sea continuum and to the unpredictability of land use and climate changes (Withers et al. 2014; Le Moal et al. 2019; Seidenfaden et al. 2022). Moreover, responses from ecosystems to different forms of nutrient input are heterogeneous and their visibility, and consequences vary with their social-ecological characteristics (Thornton et al. 2013; Levain et al. 2020). Despite the wide acknowledgment of these uncertainties, the specific analysis and treatment of uncertainty in knowledge production about eutrophication are still inchoate (Udovyk and Gilek 2013). Moreover, how the lack of knowledge relates to different ways of knowing eutrophication has only been inspected in a case of apparent lack of controversy, the Baltic Sea HELCOM strategy (Udovyk and Gilek 2013; Linke et al. 2014; Saunders et al. 2017). This case exemplifies a linear conception of science-policy interactions in eutrophication whereby science manages to ‘speak truth to power’ by neglecting uncertainties, especially those associated with contested stakeholder perceptions (Linke et al. 2014). The increasingly recognized role of intensive farming as driver of eutrophication has, however, raised calls for a more careful consideration of uncertainty, as the management of nonpoint pollution may bear significant costs for agricultural productivity and spark resistances among farmers (Paolisso and Maloney 2000; Jarvie et al. 2013; Withers et al. 2014).

This paper examines the role of knowledge claims and uncertainty in the public dispute over eutrophication in the Mar Menor lagoon (South-Eastern Spain). Intense local struggles reacting to episodes of anoxia and death of aquatic species have recently boosted the visibility of this environmental conflict and questioned the legitimacy of formal scientific advice to policy making. As we write this article, this Mediterranean lagoon became the first European ecosystem with formal legal rights thanks to a citizen-led initiative approved by Spanish authorities.^1^ New policies and governance structures are in the making, whereas perceptions over the causes and solutions to the problem differ among stakeholders (Guaita-García et al. 2022). In order to understand how public controversies are related to knowledge production and uncertainty in nonpoint pollution driven eutrophication, we draw on relational uncertainty theory (Brugnach et al. 2008, 2021) and combine the analysis of narratives with the analysis of uncertainty. We explore the key arguments in contestation, the ambiguity they bring about, how they are underpinned by knowledge claims, and how these factors are fuelled by different forms of uncertainty. Furthermore, we look for what commonalities could open avenues for dialog among contested narratives.

## Case study background

The Mar Menor is a 135 km^2^ coastal lagoon located in the Spanish Region of Murcia, host of emblematic and endangered aquatic species (see case description in the Appendix). Over five decades, the Mar Menor has been influenced by a variety of pressures from important socioeconomic changes in the area, namely, touristic promotion, rapid urbanization of the coastline, fabrication of sand beaches, expansion of ports and canals with the Mediterranean for navigation and a major transformation of the inland agricultural activity. Triggered by the construction of the Tajo-Segura water transfer, the area shifted from a structure based on family agriculture of mostly rainfed crops and cattle to a much larger area based on intensive vegetable production for exportation to European countries (Martínez Fernandez et al. 2013; Carreño 2015). This expansion attracted international food companies as well as thousands of migrants to work in the fields (Pedreño Cánovas et al. 2015). After a drought period compromised the Tajo-Segura supply in the 90 s, the Segura river basin district responsible for water management promoted the use of salted groundwater. They supported the installation of individual desalination plants all over Campo de Cartagena and built infrastructures to release residual brine into the lagoon.

Despite early warnings from scientists and environmentalists, all these changes occurred without much planning or consideration of environmental consequences. Until the lagoon’s water turned brown in the first algal bloom in 2016. Public authorities took some immediate actions. The Segura river basin district closed down desalination plants and illegalized brine-dumping to the lagoon. In addition, the Regional Government created one Scientific and one Social Participation Committee as well as an official information channel.^2^ In 2017, 19 scientists and 5 technicians from 10 different institutions produced a comprehensive assessment (Comité de Asesoramiento Científico del Mar Menor 2017) gathering the existing knowledge about the problem. Yet the purported consensus achieved in this assessment tore apart in 2018, when a share of the scientists and social organizations abandoned the committees after successive complaints about their functioning.

The second eutrophic crisis took place in October 2019 after a major flood event. An episode of euxinia: oxygen depletion triggering the toxic release of hydrogen sulfide that caused the massive death of aquatic species. These events sparked a strong social reaction as well as a political battle over the causes of the events and the competences for taking action. The environmental conflict became visible for the first time in national and international media. Yet, a third eutrophic event took place two years later in August 2021. This time was anoxia and stranding of shellfish and small fish species. The most important scientific assessments on these two episodes were produced by the Spanish Institute of Oceanography (Ruiz et al. 2020 and 2021), a public national research institute with a campus on the lagoon’s shore.

Presently, conditions in the Mar Menor are increasingly sensitive to external pressures such as floods or peaks in temperature (Ruiz et al. 2021). This is accompanied by political crises among involved public authorities (municipal, regional, and national) and regular social protests. In 2018, the Murcian Parliament passed a regional law promoted by the parties in opposition. It was soon superseded in 2020 by a second law led by the governing party. This party has governed for over 40 years and is often criticized for its connections with agroindustrial economic networks (Pedreño Cánovas et al. 2022). The new 2020 law foresees measures for adapting all productive sectors with an impact on the lagoon. Concerning agriculture, it mandates the creation of shrub corridors in every farm to prevent erosion, a new system for accounting the use of fertilizers and the ban for inorganic fertilizers in a 1500 m strip band. On its counterpart, the Ministry for Ecological Transition released two policy programs for the Mar Menor. The first one in 2017 was approved right after a shift in the government and was never implemented. The second one in October 2021, following the third eutrophic crisis, foresees a complex set of watershed-level interventions with an important budget. Finally, the new citizen legal initiative on the Mar Menor foresees the creation of a new autonomous and participatory governance structure in three committees: (i) one including representatives of national and regional administrations (6) and citizens (7); (ii) one with representatives from local administrations and social and economic sectors; and (iii) one with scientists from local and national universities and research institutions. How these new institutions are crafted and accommodated among existing ones is still to be seen.

## Theoretical framework

The analytical framework of this article is grounded in a relational approach to uncertainty as proposed by Brugnach et al. (2008). This view pays attention to uncertainty as a knowledge relationship, that is, as something that emerges from the position and the social context of knowledge holders within a given social-technical-environmental system. More specifically, uncertainty is defined as “a situation in which there is not a unique and complete understanding of a problem or a system to be managed” (Brugnach et al. 2008, p. 4). In environmental management, social actors hold diverse perspectives and knowledge of particular problems. What is knowable in a system depends as much on the existing technical and scientific capacity as on the expectations, concerns and relations between those actors that define what are the problems or issues at stake (Brugnach et al. 2021). For instance, in the past, many eutrophication problems focused on phosphorus from urban sources as the main limiting factor for the proliferation of algae blooms. It took lengthy fought battles among specific actors and tons of scientific evidence to recognize the important role nitrogen from agricultural sources played (Bourblanc 2019; Le Moal et al. 2019).

The relational uncertainty framework distinguishes between three forms of uncertainty: unpredictability, incomplete knowledge and ambiguity. Unpredictability is an ontological form of uncertainty (Walker et al. 2003) derived from the inherent complexity and variability of social-ecological dynamics. It refers to knowledge that presently *we cannot have*. Incomplete knowledge refers to epistemological uncertainty in the knowledge base, either because of lack of data, insufficient methods or theoretical understanding. Incomplete knowledge concerns what *we do not know* now but might be able to know if we enhance our analytical capacity (e.g., by doing more research, collecting more data, etc.). The distinction between these two forms of uncertainty is however situation specific. In the context of nonpoint pollution driven eutrophication, the variegated sources of nutrients may fall under incomplete knowledge if there is technical, legal and cultural possibility of tracking down leakages. However, it may well be that the number of sources is too vast to monitor, or that it is so complex that it is impossible to be measured, thereby becoming unpredictable (Whitney 2010).

Ambiguity is a third form of uncertainty related to the existence of multiple valid knowledge frames that do not overlap or are in disagreement (Brugnach et al. 2008). Ambiguity indicates disparities in actors´ knowledge and knowing. Whereas these disparities could be rooted in value differences, ambiguity is not equivalent to ambivalence or value difference (Brugnach 2017).Under conditions of ambiguity, actors have different understandings and underlying assumptions on what the problem is, and why it is a problem, and what to do about it (Giordano et al. 2017; Kovacic and Di Felice 2019). Furthermore, different problem definitions are usually tied to specific solutions or pathways in the form of narratives (Molle 2008). Compared to knowledge frames, narratives both describe and prescribe, conveying certain shared understandings of the social order that struggle to gain power or visibility (Evans et al. 2020; Di Felice et al. 2021). In this paper we follow Cabello et al. (2018) definition of narratives as stories about causality that connect the what and why of environmental problems to particular solutions, and that are often used to promote particular policy interventions and management models. In the context of public disputes over eutrophication, there are commonly competing narratives, and thus ambiguity, in the causes triggering algae blooms, as well as in the adequate measures to improve water quality or to reduce nutrient inputs (Thornton et al. 2013).

The different forms of uncertainty are not however independent. For instance the unpredictability of anoxia events bears knowledge gaps about the many factors involved and may lead to different interpretations of why exactly they happened (Mascareño et al. 2018). To analyze interrelated uncertainties, van den Hoek et al. (2014) proposed the cascades of uncertainty methodology. Adapting the theory of cascades from climate change literature, this framework identifies the above described forms of uncertainty together with interconnections between them across three domains: ecosystem, society and technology. The separation between domains obeys analytical purposes as it eases visualization of interrelations and interpretation. A description of the cascades methodology is provided in the Appendix. In the next section we describe the process followed for data collection and analysis.

## Materials and Methods

**Fig. 1:**
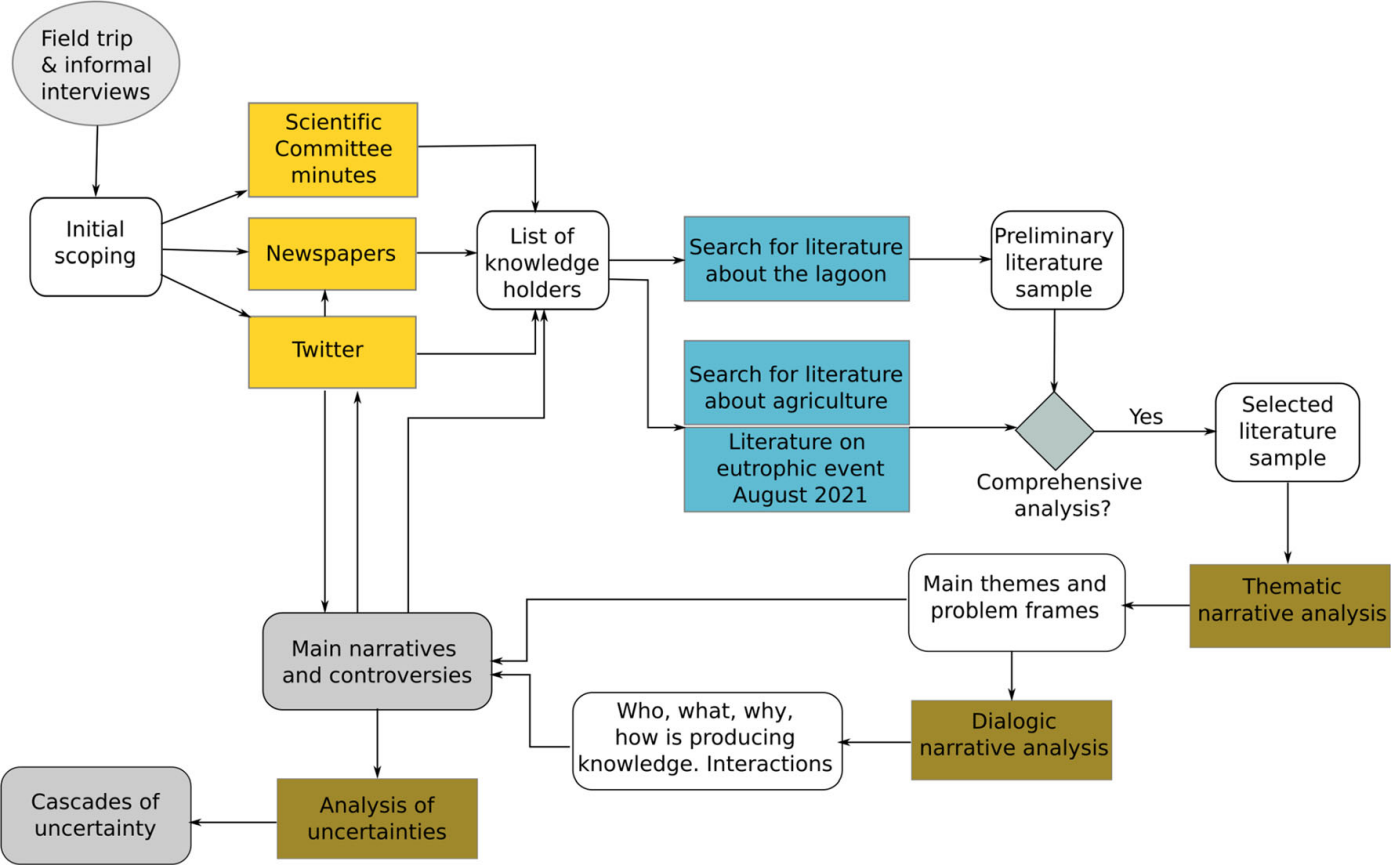
Flow chart of qualitative analysis process. Colored boxes represent iterative analytical steps as outlined in this section. White boxes represent interim analytical outputs. Gray boxes are the two final outputs

This paper builds upon qualitative analysis of public media and a sample of scientific and gray literature. The literature has been authored by scientists and other relevant actors (both individuals and organizations) that (1) actively produce knowledge about the eutrophication of the Mar Menor and (2) are echoed by local and social media, thereby contributing to public debates. The data gathering and analysis process was organized in several iterative steps (Fig. 1).

Identification of actors producing context-specific knowledge. To both gain an initial understanding of the Mar Menor context and identify knowledge holders, we first spent 5 days of fieldwork visiting different actors and ran 6 informal interviews with researchers from local institutions in March 2021. Second, we followed all Twitter accounts that tweeted with active hashtags (#MarMenor; #SOSMarMenor), identifying both news and actors related to knowledge generation. In parallel, we selected the three most read regional newspapers with different ideological orientations (La Opinión de Murcia; La Verdad; Eldiario.es/Murcia) and revised over 200 articles containing knowledge claims about the Mar Menor eutrophication, all published during 2021. Finally, we reviewed the minutes from meetings of the Mar Menor Scientific Committee^3^ and analyzed the composition of its working groups.

Selection of literature. The previous process gave us a preliminary overview of the main knowledge and uncertainty claims as well as their related controversies in public media. It also provided an initial list of knowledge holders together with key literature that was collected from public websites and scientific journals. From this preliminary sample, we selected those studies that explicitly addressed a comprehensive analysis of the eutrophication problem, considering its causes and pointing at potential solutions. As the analysis advanced and we gained a deeper understanding of the main controversies, we expanded the list of knowledge holders and the sample of literature in order to include standpoints from the agricultural sector. After the third eutrophic crisis started in August 2021, new public controversies emerged together with explanatory reports and policies. We included three new documents to cover the episode. The selected literature sample includes 32 documents (Table SA1 in the Appendix): scientific papers (3), PhD dissertations (1), scientific reports (12), reports from environmental (4) and agricultural (1) organizations, reports from public and private consultancies (3), transcripts from experts talks (5) and policy documents (3). Dates of publication range from 2013 to October 2021 when the ‘Framework on Priority Actions on the Mar Menor’ from the Spanish Ministry for Ecological Transition was released. The final list of publicly relevant knowledge holders (institutions and organizations) is shown in Table SA2 in the Appendix.

Narrative analysis. The selected literature was coded in QualCoder open source software for qualitative data analysis. The process followed a mixed deductive and inductive approach in two iterative steps. In order to further explore public controversies around the Mar Menor eutrophication process, we developed a two-stage analysis of narratives. The first stage involved a thematic analysis to examine the content of the narratives in terms of how authors framed the constellation of issues around the eutrophication problem and its solutions (Allen 2017, p. 1069; see thematic narratives in Table A5 in the Appendix). We also explored how they framed problems of the agricultural sector in relation to the lagoon’s situation. The second step focussed on dialogic narrative analysis to further examine relations between identified themes as well as the authors involved and their position in the wider context of environmental conflict in the Mar Menor (Allen 2017, p. 1070; see dialogic narratives in Table A5 in the Appendix). In this vein, we analyzed the process of knowledge production in terms of the who, what, why and how was writing about specific themes. We also analyzed how authors interacted by building upon or contesting each other in their published works (using explicit references). Altogether, these analyses enabled delineating two broad narratives with a set of key arguments in contestation. To triangulate these findings, we qualitatively analyzed Twitter discussions around identified controversial themes from Oct 2021 to June 2022 (see Table A3 in the Appendix for the list of Twitter threads).

Analysis of uncertainty. Following the above described cascades methodology (van den Hoek et al. 2014), we iterated the coding process in order to explore interrelated uncertainties. We departed from identified controversies that represent ambiguities and examined how other forms of uncertainty connected to each controversial theme. We identified explicit mentions of incomplete knowledge and of those aspects deemed unpredictable. We further categorized each uncertainty claim as pertaining to the ‘ecosystem,’ ‘technology,’ ‘society’ domains, or to any of their intersections (see description of Figure SA1 in the Appendix). The final outputs of this process are four double-entry matrices, one per controversial theme, with claims classified by type of uncertainty and by analytical domain (see an example in Table A4 in the Appendix). The cascades template (Figure SA2 in the Appendix) was used to display each set of interconnected uncertainties across the different domains of the social-technical-environmental system.

**Fig. 2:**
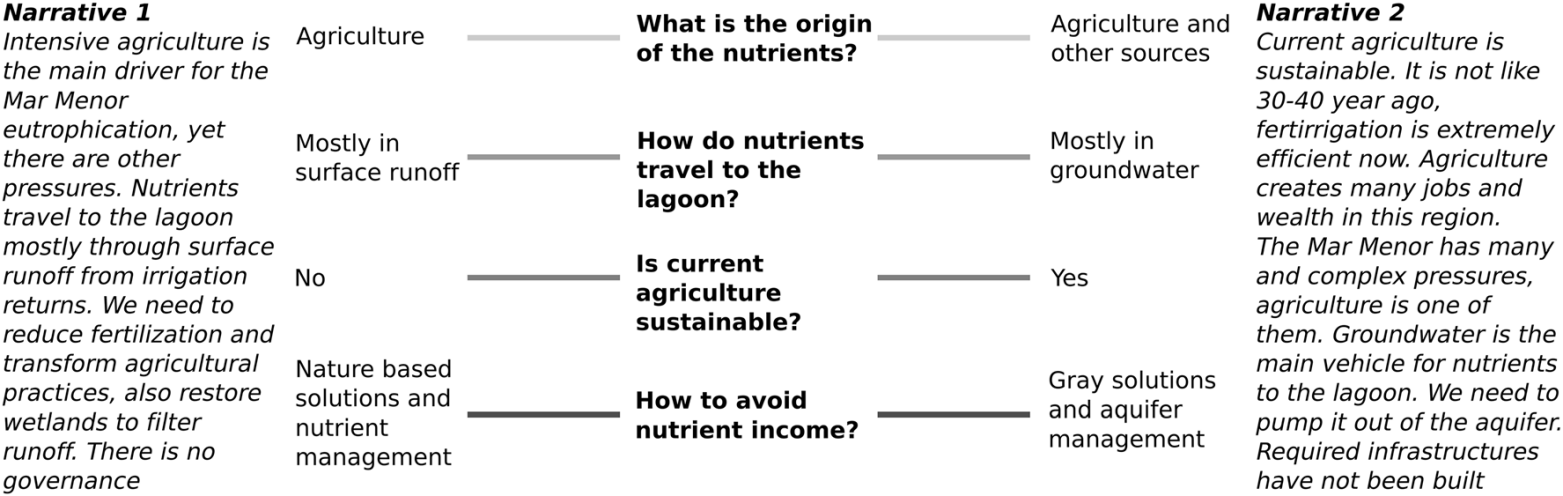
Summary of arguments in controversy and contested narratives

## Results

### Contested narratives about eutrophication

The first overview of the collected literature reveals an intense knowledge production on the Mar Menor degradation since the 70 s. Among the reviewed documents, there is a manifested dominance of biophysical sciences, while social sciences and humanities are underrepresented. Disciplines addressing the eutrophication process include aquatic ecology, biology, hydrology, and hydrogeology, followed by soil sciences and engineering about mines and water decontamination. In the literature about agriculture and its relation to the lagoon, agronomy and civil engineering play an important role, whereas sociology and environmental economics are also disciplines discussing the sustainability of farming activities.

The analysis of interactions between authors reveals a dynamic of growing polarization. Agreement interactions predominated in the revised literature until the scientific assessment of 2017. Contestations between authors emerged in 2018, when the Scientific Committee broke down, and augmented in number until 2020. In addition, we observe an increased sophistication in the analyses of the complex ecological processes involved in the lagoon eutrophication, especially after the 2019 anoxic event, with different authors providing slightly different explanations of the phenomena (see Ruiz et al. 2020, 2021 and Pérez-Ruzafa 2020, 2021a, b). Finally, the literature about agriculture produced after 2018 attests of a defensive reaction against the increasing appointment to intensive agriculture as the main source for nutrient overenrichment of lagoon’s water (Fundación Ingenio 2020; Aledo et al. 2021).

Overall, the line-up and contestation of arguments between authors depict two main clusters representing opposing narratives (Fig. 2). Several controversies add up to conform two coherent sets of claims that fundamentally differ in their answer to four interconnected questions. First, whether current agriculture is the main source for nutrient accumulation in the lagoon, or if there are multiple nutrient sources with no exact weight appointed. Second, whether the main vehicle for nutrients to travel to the lagoon is surface runoff from irrigation returns, or nitrogen-full groundwater accumulated during previous decades of inefficient irrigation. Third, whether current agricultural practices are sustainable or not, and in what terms. Finally, and following previous lines of reasoning, what are the adequate measures to prevent nutrient leakage to the lagoon.

There are, however, commonalities. All authors acknowledge the existence of a long-term process of eutrophication in the Mar Menor, caused by nutrient enrichment in the last decades (see for instance the different chapters of the Scientific Assessment authored by different teams of scientists and technicians, Comité de Asesoramiento Científico del Mar Menor 2017). In this sense, the ecological scientific knowledge describing the phenomena is not questioned. They further agree on the need for reducing nutrient discharges and the many other pressures received by the lagoon. For instance, most mention heavy metal leakage from Southern sierras as a side-pressure debilitating the ecosystem. There is also agreement on the increasing flood problem dragging massive amounts of sediments and fertilizers into the lagoon. Moreover, there is convergence of arguments about the complexity of the situation and the impossibility of a single solution.

Perhaps, the strongest commonality we observed is the agreement on the lack of effective public action. The issue of governance and responsibility becomes recurrent in both narratives after 2018 (see for instance the successive calls for stronger governance in the monitoring reports signed by the president of the Scientific Committee, Pérez Ruzafa 2020, 2021a, b). The succession and superpositions of plans and figures of protection had not yet manifested in visible changes for the ecosystem. One discussed reason for this stagnation is that the question of who is responsible for action is part of the controversy. If solutions come in hand of managing the aquifer by extracting and denitrifying groundwater, then the Segura river basin district and the Spanish Government are the responsible public bodies. If they are tied to the management of nutrients through fertilization practices, then it is the Regional Government of Murcia in charge. The idea of a drastic change in agricultural productivity is at odds with the regional alignment in defense of agricultural sustainability. If political stall is underpinned by contestation over the causes and solutions to the eutrophication problem, then the existence of uncertainties over those causes and solutions can only reinforce controversies and delay action (Karlsson and Gilek 2020).

### Cascades of uncertainty in a nonpoint pollution dilemma

Mentions of uncertainty abound across the reviewed literature. The two largest scientific assessments (Comité de Asesoramiento Científico del Mar Menor 2017 and Ruiz et al. 2020) provide descriptions of existing gaps of knowledge and needs for further research. Paradoxically, these authors insist on how much is already known about the lagoon ecology but at the same time, how much it is still ignored. Moreover, acknowledgment of uncertainties in data and applied methods are common across scientific works on the lagoon. Mentions of unpredictability are less frequent and mostly refer to certain aspects that are difficult to know either because they are too complex, because they refer to past processes with no recorded data or because they are contextual changes out of local influence. In what follows, we unpack the interconnections between these uncertainties and the four controversial themes depicted in Fig. 2.

**Fig. 3:**
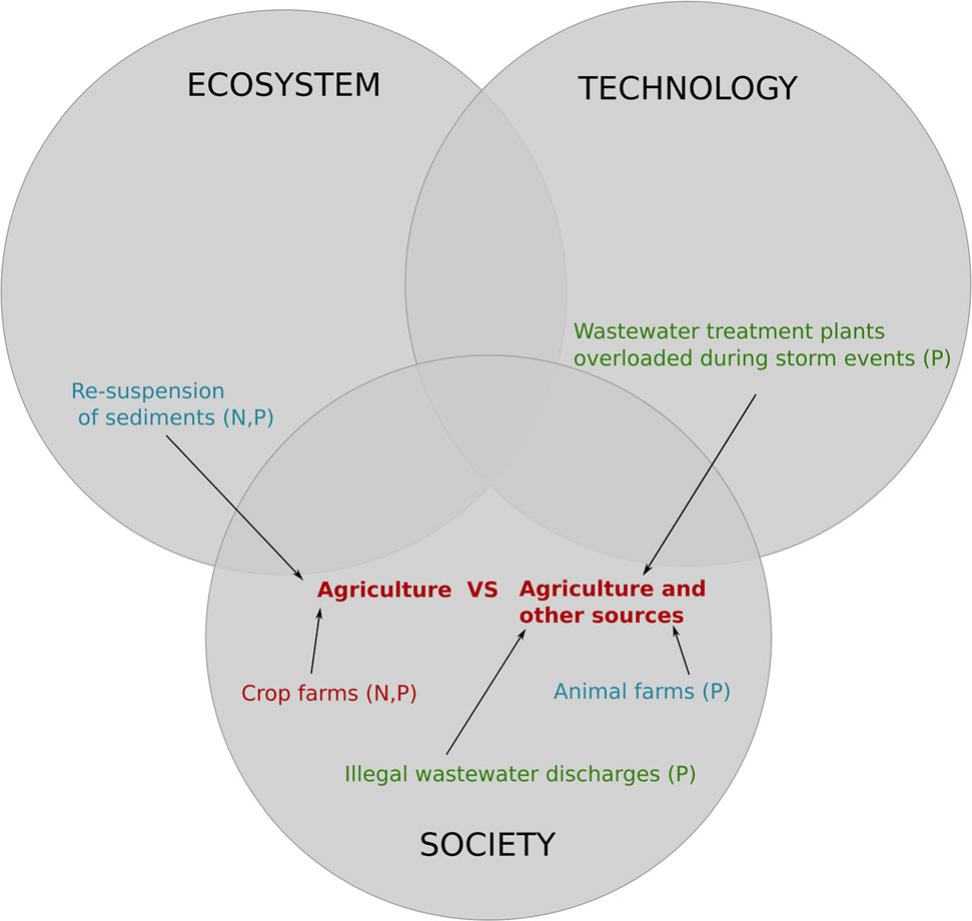
Cascade 1—What is the origin of the nutrients? Color code: Red refers to ambiguities; Blue refers to incomplete knowledge; Green refers to unpredictability

**What is the origin of the nutrients?** The first cascade (Fig. 3) connects the ambiguity observed regarding the weight of agriculture as the main driver for the Mar Menor eutrophication with the insufficient knowledge and the unpredictability about other sources of nutrients. Contrary to other cases in the literature (Bourblanc 2019; Saunders et al. 2017), we found no dispute over which nutrient is the so-called limiting factor. Authors in the Mar Menor acknowledge that the lagoon receives both nitrogen and phosphorus and they alternate on the limiting role depending on a number of conditions (a recent explanation is provided in Fernández-Alías et al. 2022). There is unanimity on past urban wastewater dumped to the lagoon as a major source of phosphorus. However, the current origin of this nutrient is unknown. Reports from the Spanish Institute of Oceanography hypothesize it may come in a fraction from eroded soils while also resuspends from lagoon’s sediments where it has settled over decades (Álvarez Rogel et al. 2017; Ruíz et al., 2020). In addition, many studies emphasize the insufficient capacity for wastewater treatment in specific locations, seasons and storm events (see for instance Álvarez Rogel et al 2017; Faz Cano et al. 2017; Ruzafa 2021a and b). Moreover, the contribution from dysfunctional waste treatment in the growing pig farm industry is seen as undetermined. A stronger consensus is observed around the large area of intensive vegetable crop farming as the main source of nitrogen in the form of nitrates from fertilizers.

**Fig. 4:**
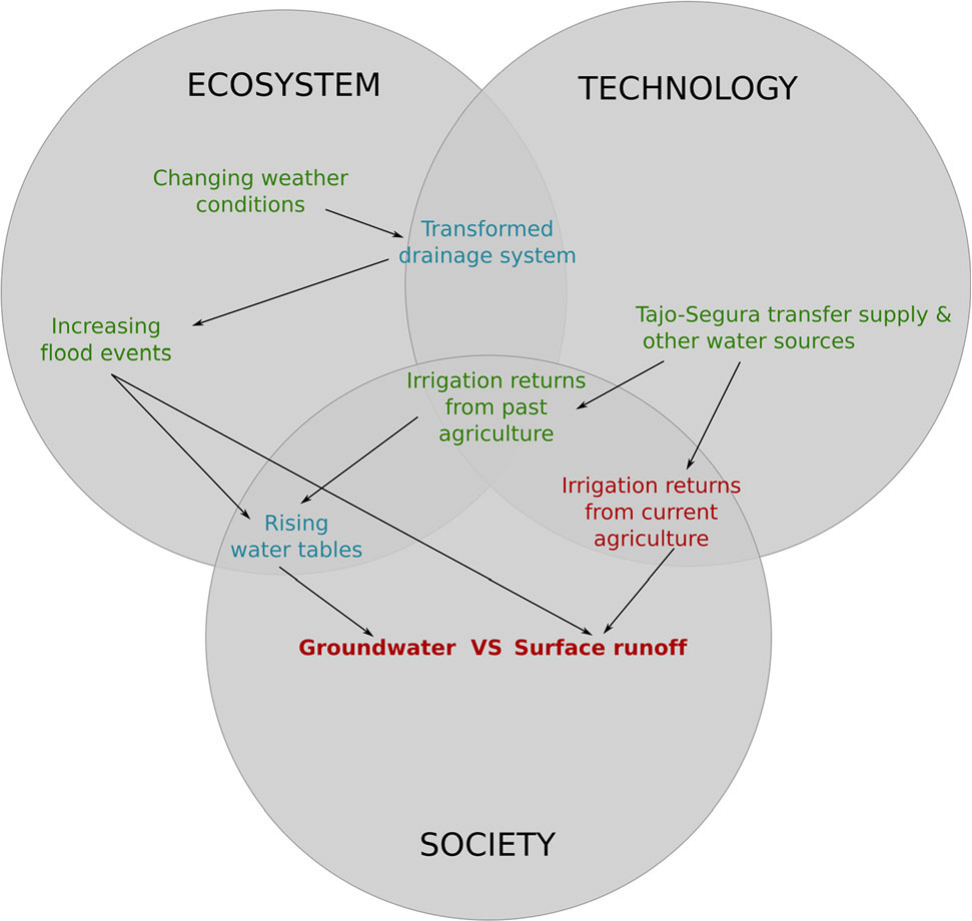
Cascade 2—How do nutrients travel to the lagoon? Color code: Red refers to ambiguities; Blue refers to incomplete knowledge; Green refers to unpredictability

**How do nutrients travel to the lagoon?** The avenues nitrogen follows from agricultural fields to the Mar Menor are strongly contested between ‘mostly surface runoff’ or ‘mostly groundwater’ (Fig. 4). Some authors from the hydrogeology field have strived to position the role of the shallow aquifer as the main vehicle for water-nitrogen flows (Jiménez-Martínez et al. 2016). They emphasize the lack of reliable data and insufficient knowledge about groundwater dynamics. These pro-aquifer arguments are uptaken by agricultural organizations defending the sustainability of current agricultural practices (Narrative 2, see Fundación Ingenio 2020). In their narrative, the water accumulated in the aquifer comes from past agricultural practices before the technological transition to drip irrigation in the 90 s and slowly travels to the lagoon since then. Yet it is impossible to know how much water has accumulated and when because of the lack of historical records. Authors opposing this narrative contend that nitrogen flows to the lagoon mostly through surface runoff from present day irrigation returns, fed by a variable volume of Tajo-Segura water mixed with other water sources (Martinez-Fernandez 2013; Martínez and Esteve-Selma 2020). Underpinning this ambiguity, there are different quantitative models showing different figures for the relative weight of surface and groundwater flows to the lagoon (Jiménez-Martínez et al. 2016; Contreras et al. 2017; TRAGSA 2020; more recently Senent-Aparicio et al. 2021).

**Fig. 5:**
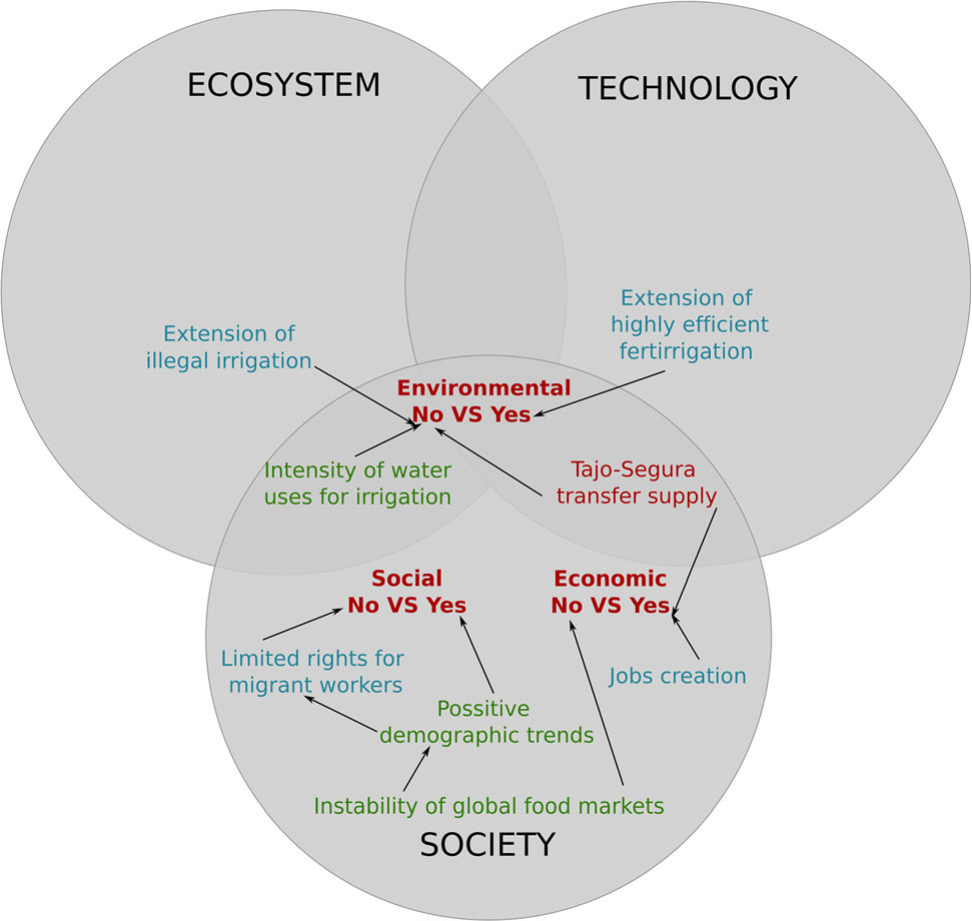
Cascade 3—Is current agriculture sustainable? Color code: Red refers to ambiguities; Blue refers to incomplete knowledge; Green refers to unpredictability

The aquifer controversy is deeply political because it is connected to the defense of a particular set of solutions focussed on groundwater management (Cascade 4, Fig. 5). Ever since individual desalination plants were closed down by the Segura river basin district in 2018, groundwater levels started to rise. The cause for groundwater level rise is also contested. Hydrogeologists link it to the increasing number and intensity of flood events diminishing the role of irrigation returns (García-Aróstegui 2021). Flood events are indeed a major trigger for eutrophic crises in the lagoon. The expansion of agricultural land has occupied dredging canals thereby boosting runoff and erosion during intense rain episodes. This part of the cascade connects the unpredictability of climate change and flood events with the incomplete knowledge about the situation of the drainage system and about how floods ultimately increase both surface and groundwater water-nitrates flows to the lagoon..

**Is current agriculture sustainable?** As the controversy over the role of agriculture in the eutrophication of the Mar Menor grew, a new discussion emerged around the sustainability of this productive sector (Aledo et al. 2021). The discussion is framed using the three classic dimensions of sustainability: environmental, social and economic (Fig. 5). In this vein, ambiguity surrounds the meaning and relative importance of each of these criteria. In the pro-agriculture narrative, farming practices are environmentally sustainable because they use hyper efficient ferti-irrigation systems. Efficiency in nutrient and water use is being further enhanced by the adoption of digital tools to monitor the exact crop needs. In this narrative, there are no irrigation returns nor release of fertilizers in present day farms. However, the scale and rate of expansion of these technologies across Campo de Cartagena is currently unknown.

Contesting claims to the environmental sustainability of farming practices refer to the unknown extension of illegal irrigation and to the intensive water allocation per hectare to sustain an unknown number of crop yields (García-Moreno et al. 2018; Greenpeace 2021). The Tajo-Segura water transfer is another contesting argument. On the one hand, the transfer generates environmental impacts and social conflicts in the donor Tajo river basin. On the other hand, the good quality of Tajo’s water is claimed to be fundamental for the economic sustainability of the agricultural sector. The rest of water sources face severe quality problems that may compromise productivity in the long run.

In the midst of this disputed backdrop, the discussion on the meaning of sustainability is expanded to the social and economic importance of the agricultural sector in the Murcia region. The process of intensification of crop production, coupled to the internationalization of food supply chains, created thousands of jobs for farm workers that were occupied by immigrants from Africa and Latin America (Pedreño Cánovas et al. 2015; Aledo 2021). The attraction of new settlers together with their families shifted regressive demographic trends. Furthermore, the agricultural sector has flourished in an important number of industries in charge of post-harvest processing and direct exportation to European retailers. This model however faces the unpredictability of international food markets while resting on unjust labor conditions for migrant workers (Pedreño Cánovas et al. 2015; Aledo 2021).

**Fig. 6:**
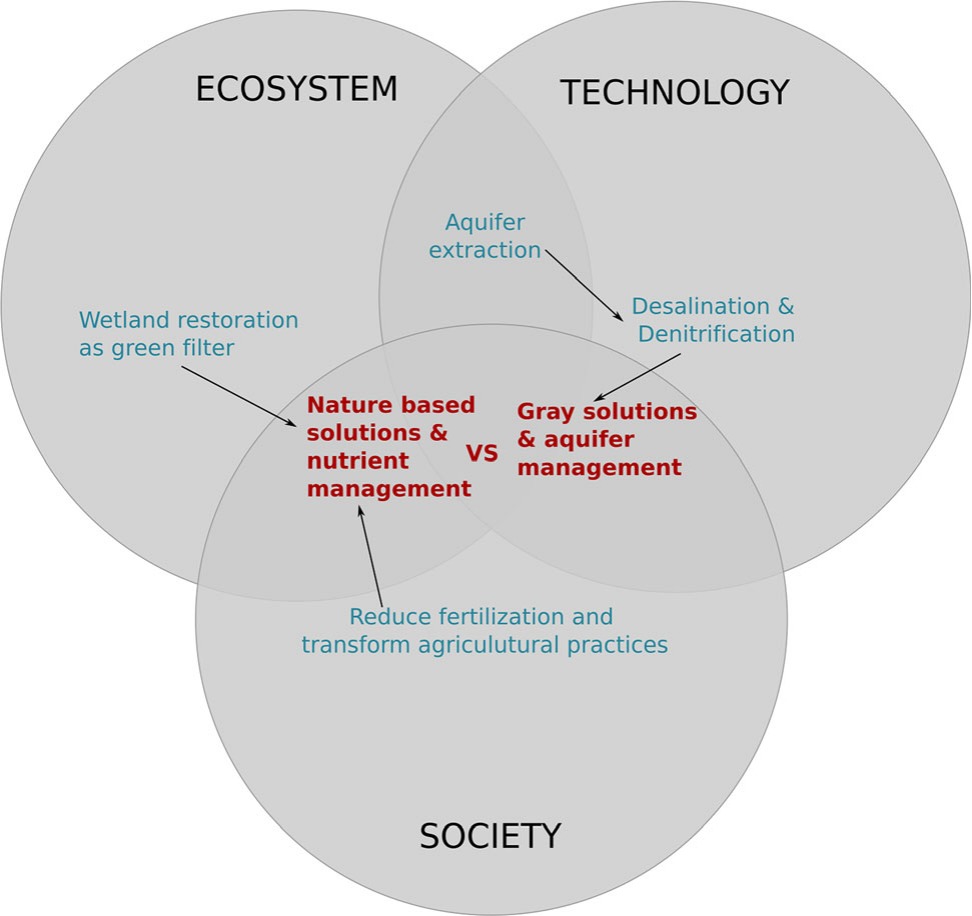
Cascade 4—How to avoid nutrient income to the lagoon? Color code: Red refers to ambiguities; Blue refers to incomplete knowledge; Green refers to unpredictability. Note that overlapping areas have been enlarged to fit the ambiguity about solutions in the central area

**How to avoid nutrient input to the lagoon?** This cascade is the ultimate step in the causal chain of ambiguities, where the previous coalesce in the question of how to cope with the eutrophication problem (Fig. 6). The constellation of proposed pathways is large and complex (see SOLUTIONS in Codebook, Table A5), as all authors agree that there is no silver bullet for the Mar Menor. Our analysis is restricted to the means for reaching the popularized ‘zero discharge’ target. In this regard, one narrative defends the transformation of agricultural practices to reduce fertilization which is considered the ultimate cause of the problem (see for instance García-Moreno et al., 2018; Martínez and Esteve-Selma 2020). It also claims for the restoration of wetlands to act as filters for surface water flows (Ecologistas en Acción, 2021). The other narrative focuses on managing the aquifer as the centerpiece for nutrient leakage to the lagoon, which is assumed to be growing in correlation to the rise of groundwater levels (see for instance Jiménez-Martínez et al. 2016; Fundación Ingenio 2020; García-Aróstegui 2021). This solution is operationalized through an infrastructural complex of groundwater pumping, desalobration to achieve irrigation quality and further denitrification to release nitrogen-free brine to the Mediterranean (branded as the ‘Zero Discharge Plan’, Ministerio para la Transición Ecológica y el Reto Demográfico 2019).

The controversy around the two type of interventions is illustrated in terminological discussions about whether the crux of the matter is the management of ‘water flows’ or of ‘nutrient flows’, whether the intervention should be over the ‘origin’ of water-nutrient flows or over the ‘destiny,’ whether effective solutions are ‘nature-based’ or ‘tube-based.’ As we discuss below, these dichotomies echo larger confrontations in water and agricultural governance in Spain. Moreover, they convey essentially divergent visions on how agricultural production should be (Evans et al. 2020). Yet, as our analysis has shown, they both lie on a set of assumptions while facing important unknowns.

## Discussion

The disrupted ecosystem of the Mar Menor lagoon has become the frontline for environmental politics in Spain. The Mar Menor question remobilizes long-standing disputes between a tradition of large water infrastructures for agricultural development and new environmental discourses and policies (Swyngedouw and Williams 2016; del Moral et al. 2017). It also epitomizes the question of centralized versus decentralized water governance in Spain (Del Moral and Do Ó, 2014) and that of coherence between water and agricultural policies (Cabello and Madrid 2014). Yet, this background alone is insufficient to understand the magnitude of an environmental conflict that spans way beyond the local, leading to grant legal rights to an ecosystem for the first time in Europe. Inter and transdisciplinary research is still needed to explore the cultural transformations brought by the high visibility of the dead fishes laying at the lagoon's shore. As Levain et al. (2020) put it, those ‘noisy’ eutrophication cases that gain enough social visibility have been successful in mobilizing interdisciplinary approaches capable of addressing the social roots and consequences of aquatic overfertilization. This paper contributes to such efforts by unraveling the public dispute over the Mar Menor eutrophication.

Our results show two increasingly polarized narratives that fundamentally deviate in the causes and sources for nutrient enrichment and in the type of solutions seen as effective, all of which relate to contested visions on agricultural sustainability. In fact, the core of the dispute can be reduced to a deep cultural confrontation about the meaning of agricultural intensification as discussed in Evans et al. (2020). Agricultural development and internationalization have been the cornerstone of Murcia’s public policies for decades. It has also been a key element in the construction of a strong identity of farmers as innovators and entrepreneurs (Pedreño Cánovas et al. 2022). This role has been challenged for the first time by accusations of intensive farming as a polluting activity. In turn, the framing of farmers as polluters sparked a shielding reaction among authors close to agricultural organizations. To counter the growing perception that they do not care for the lagoon’s situation, farmer environmentalism (Paolisso and Maloney 2007) is claimed in the form of commitment to zero nutrient discharges through irrigation efficiency, and coupled with the social and economic dimensions of their activity.

Similarly polarized narratives shaped by the degree of affinity to farming activities have been described in eutrophication cases in the Chesapeake Bay (Paolisso 1999; Paolisso and Maloney 2007), Chiloé Island (Mascareño et al. 2018) and Brittany (Le Chêne 2012; Levain et al. 2020). These cases share with the Mar Menor a rapid shift from rural areas to become nodes in transnational food supply networks. In turn, the insertion in global chains heavily restricts production criteria while increasing interdependencies and bureaucracy for farmers in a private governance system (Paolisso 1999; Pedreño Cánovas et al. 2022). Paolisso and Maloney, (2000) describe farmers’ moral reactions against nutrient control regulations in Maryland that limited their already constrained autonomy while not being sufficiently justified by scientific evidence. As our analysis with the cascades revealed, uncertainty plays different roles in the current dynamics of narrative contestation in the Mar Menor.

First, we observed that certain knowledge gaps play a major role in disputing the centrality of agricultural responsibility. Overall, there is incomplete knowledge about the socio-hydrological behavior of the profoundly modified watershed of the Mar Menor. Actual uses of water and fertilizers are unknown. It is also unknown how much of daily ferti-irrigation is uptaken by crops and how much returns to the system. Crucially, there is both incomplete knowledge and unpredictability related to the variety of sources for nitrogen and phosphorus that steer algae blooms. Second, we found a reinforcing feedback between the scientific battle over the role of the aquifer as a vehicle for water-nutrients flows and the political battle over the adequate solutions to reduce such flows. As discussed by Withers et al. (2014), the temporality of nutrient leakage (legacy of slow groundwater versus rapid runoff from daily irrigation and floods) appears as a key uncertainty, a mix of incomplete knowledge of present day farming practices and unpredictability due to the lack of past records. Third, new knowledge (data, models) is continuously mobilized by both narratives to address those uncertainties that help them to claim credibility and legitimacy, ultimately contributing to polarization.

These insights speak to the idea that the difficulty in, and the consequences of, placing responsibility lie at the heart of the social complexity of eutrophication and nonpoint pollution dilemmas (Whitney 2010; Freitag 2014; Levain et al. 2020). As Amy Freitag (2014, p. 332) points out in her study on ways of knowing water quality, ‘defining responsibility entails delineating responsible actors in the system,’ which is extremely challenging in downstream systems with multi-scale interdependencies like the Mar Menor. Still, the focus on blame and responsibility stays in a narrowed problem definition where each actor, each farmer, is accused of performing a particular role. In this view, the issue is how to produce enough evidence so that uncertainties can be overcome, roles delineated, and decisions made. Brugnach et al. (2011) discuss how invoking scientific evidence is only one of the many ways in which ambiguity can be arbitrated. In fact, this strategy often entails imposing a narrative that is ‘right’ over the other that is ‘wrong,’ excluding the concerns, values, and knowledge of the disregarded narrative (Giampietro and Bukkens 2022). The new scientific committee foreseen under the legal personhood initiative will need to mobilize confronted knowledge holders in order to enlighten some of the existent uncertainties. Moreover, policy-makers and stakeholders participating in the other committees will face a value confrontation around the sustainability of agriculture. Transforming the current socio-political polarization trend may require alternative strategies for coping with the chain of interrelated uncertainties shown in our analysis (Jarvie et al. 2013; Saunders et al. 2017; Brugnach 2017).

## Conclusion

This research is part of the groundwork for initiating a knowledge co-creation process in the Mar Menor. In addition to providing a broad overview of the state-of-the-art of the literature, it helped to shed light over the core terms of the dispute and to unravel the role different types of uncertainties played in it. Finally, it helped reveal commonalities that may create opportunities for dialog and collaboration. Whereas described narratives differ in the why and how of the lagoon’s nutrient enrichment, they agree in the what. In this sense, the Mar Menor problem is described as one of eutrophication due to a rapid transformation of the lagoon’s territory at the time as one of insufficient public governance. Rather than striving for clarity on who is to blame, our future research will focus on how to share responsibility and collectively unpack uncertainties in the complex task of revitalizing the Mar Menor.

## Supplementary Information

Below is the link to the electronic supplementary material.

Supplementary file1 (PDF 1043 KB)

## Footnotes

1 The law was passed by the Parliament in April 2022, then by the Senate in September and finally released in October. More information at: https://ilpmarmenor.org/.

2 http://canalmarmenor.org.

3 https://canalmarmenor.carm.es/ciencia/comite-de-asesoramiento-cientifico/
