# Supplementary material for: Whose waters, whose nutrients? Knowledge, uncertainty, and controversy over eutrophication in the Mar Menor
Source: Ambio. 2023 Apr 7;52(6):1112–24. doi: 10.1007/s13280-023-01846-z (PMC10160264; doi:10.1007/s13280-023-01846-z)
Supplement: Supplementary file 1 — Supplementary file1 (PDF 1043 KB) [file 13280_2023_1846_MOESM1_ESM.pdf]

Ambio

Electronic Supplementary Material

This supplementary material has not been peer reviewed.

Title: **Whose waters, whose nutrients? knowledge, uncertainty and controversy over eutrophication in the Mar Menor**

Authors: Violeta Cabello, Marcela Brugnach

Introduction to the cascades of uncertainty methodology

The cascades methodology (Figure A1 below, see van den Hoek et al. 2014 in References for further explanation) represents in a figure three different forms of uncertainty together with their interconnections. Black arrows express relations between uncertainties, whereas colors indicate if the uncertainty dominantly concerns unpredictability (green), incomplete knowledge (blue) or ambiguity (red). The uncertainties and connections are displayed in different areas (1-7) depending on the domains of the system to which they are attributed. Uncertainties in the areas 1, 2 and 3 mainly concern a single domain, either the natural, technical or social one. For instance, ambiguity is always in the area of 'society' as it concerns misunderstandings or contested forms of knowledge among actors in the system. Uncertainties in the areas 4, 5 and 6 are described as hybrids between domains. For instance, the impact of a particular technology on the functioning of ecosystems may be not fully known (e.g., impact of desalination of water for irrigation on the water quality of nearby lagoon; hence in area 4), the effects of a technological solution may be contested at a societal level (e.g., if high precision irrigation for agriculture is or not sustainble; hence in area 5) or unpredictable changes in the natural system affecting human behavior (e.g., tipping points in the process of aquatic eutrophication and their effects in activities like fishing or recreation; hence in area 6). Finally, some uncertainties can concern all domains (area 7). For example, uncertainty about which strategies to apply to reduce nonpoint nutrient leakage has implications in all technological, ecosystem and societal spheres (hence classify in area 7).

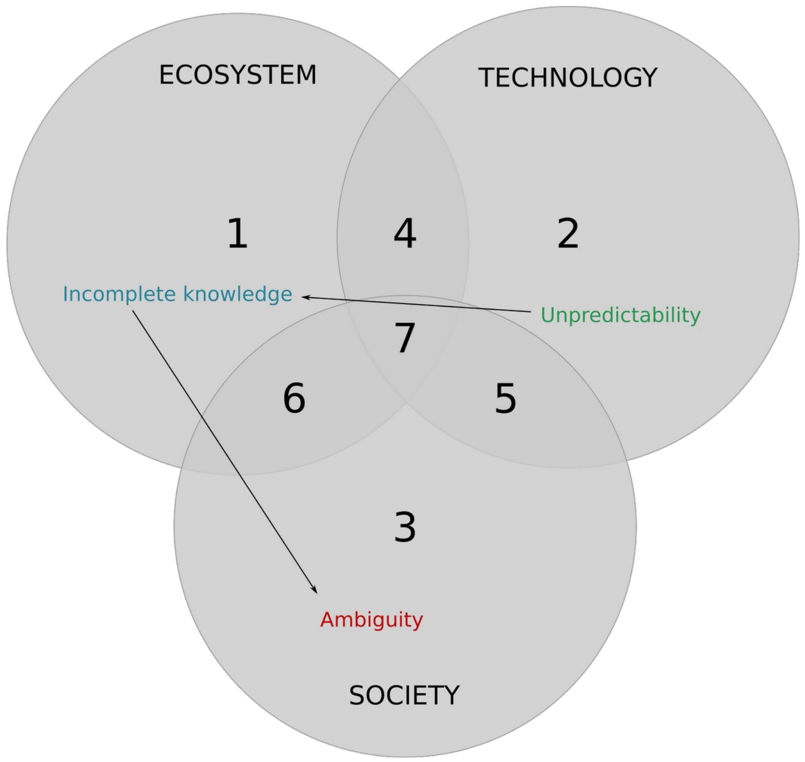

Figure A1. The Cascades of Uncertainty methodology. Source: van den Hoek et al. 2014

Introduction to the Mar Menor context

The Mar Menor is a coastal lagoon located in the Region of Murcia (SE Spain, Figure A2). Its shallow and hypersaline conditions make it a unique and diverse ecosystem of 135 km2, host of emblematic and endangered aquatic species. It is connected with the Mediterranean Sea by three canals in its East-delimiting and heavily urbanized sandbar named La Manga. Hydrologically, it is located in a closed watershed with intermittent water courses and four layers of aquifers, part of the Segura river basin district. Climatically, the area is sub-desert Mediterranean characterized by high temperatures (annual average of 15-17ºC) and low rainfall (around 300 mm per year) concentrated in few intense episodes that generate important avenues. Geographically, the lagoon’s watershed includes a vast agricultural area known as Campo de Cartagena, inland rural communities as well as coastal communities living on tourism and a residual fishing activity. Water flows in the Mar Menor watershed are intricate and mostly unaccounted for. In addition to groundwater and surface water from the Tajo-Segura basin, there are a number of desalination and wastewater reclamation plants delivering water to agriculture, the largest water consumer.

Governance responsibilities relevant for the Mar Menor eutrophication process are distributed among institutions at different levels. The Ministry for Ecological

Transition of the Spanish Government runs the Segura river basin district. It is responsible for surface and groundwater management, including the Tajo-Segura water transfer, as well as flood prevention and coastal management. Additionally, there are two large irrigation communities in charge of ensuring water supply to agriculture from external transfers and variegated internal sources (desalination, wastewater reuse, groundwater). On the other hand, land use and agricultural governance is responsibility of the Regional Government of Murcia. Local councils are in charge of urban planning and wastewater treatment. The new citizen legal initiative on the Mar Menor personhood foresees the creation of a new autonomous and participatory governance structure that will require coordination with all mentioned institutions to guarantee the conservation of the lagoon.

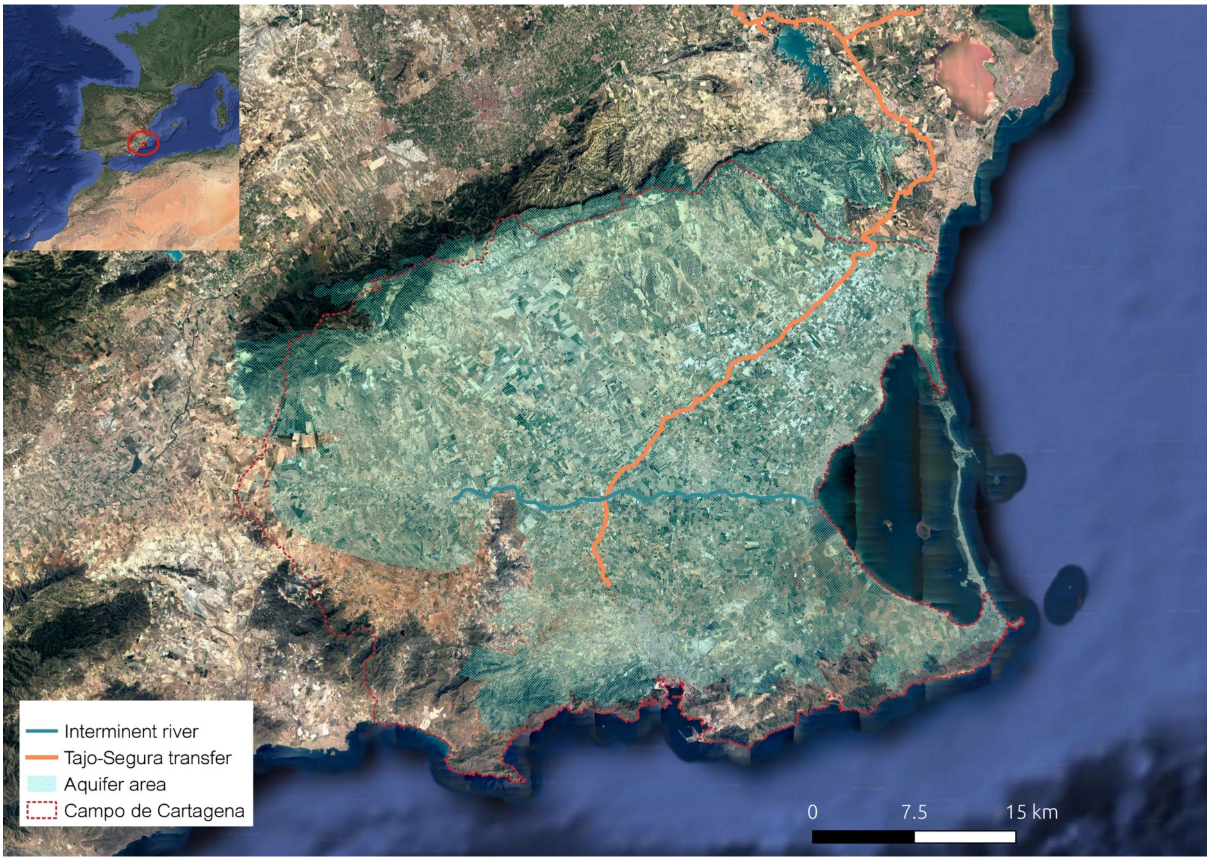

Figure A2 - The Mar Menor location in Campo de Cartagena

Table A1. Reviewed literature

Authors	Name	Date	Type	Source
J. Martínez Fernández, C. Fitz , M.A. Esteve Selma, N. Guaita , J. Martínez- López	Modelización del efecto de los cambios de uso del suelo sobre los flujos de nutrientes en cuencas agrícolas costeras: el caso del Mar Menor	2013	Scientific paper	DOI: 10.7818/ ECOS.2013.22-3.12
M.F. Carreño Fructuoso	Seguimiento de los Cambios de Usos y su Influencia en las Comunidades y Hábitats Naturales en la Cuenca del Mar Menor, 1988-2009, con el Uso de SIG y Teledetección	2015	PhD dissertation	https:// dialnet.unirioja.es/ servlet/tesis? codigo=100840
A. Pedreño, C. de Castro, E. Gadea, N. Moraes	Sustainability, resilience and agency in intensive agricultural enclaves	2015	Scientific paper	DOI: 10.4422/ager.2015. 02
J. Jiménez- Martínez, J.L. García-Aróstegui, J.E. Hunink, S. Contreras, P. Baudron, L. Candela	The role of groundwater in highly human-modified hydrosystems: a review of impacts and mitigation options in the Campo de Cartagena-Mar Menor coastal plain	2016	Scientific review paper	DOI: dx.doi.org/ 10.1139/er-2015- 0089
M.F. Gimenez Casalduero,C. Marcos Diego, F.J. Oliva Paterna, A. Pérez Ruzafa, F. Robledano Aymerich, M.M Torralva Forero	Informe integral sobre el estado ecológico del Mar Menor. ECOLOGÍA LAGUNAR	2017	Scientific report	https:// canalmarmenor.car m.es/wp-content/ uploads/2020/07/ Informe-Integral- sobre-el-estado- ecol%C3%B3gico- del-Mar-Menor.pdf
J.L. García-Aróstegui, F. Marín Arnaldos, D. Martínez Vicente	Informe integral sobre el estado ecológico del Mar Menor. HIDROGEOLOGÍA	2017	Scientific report	https:// canalmarmenor.car m.es/wp-content/ uploads/2020/07/ Informe-Integral- sobre-el-estado- ecol%C3%B3gico- del-Mar-Menor.pdf
J. Álvarez Rogel, M.A. Esteve Selma, A. Faz Cano, R. Gómez Cerezo, V.M. León León, J. Martínez Fernández, M.J. Martínez Sánchez	Informe integral sobre el estado ecológico del Mar Menor. CUENCA VERTIENTE	2017	Scientific report	https:// canalmarmenor.car m.es/wp-content/ uploads/2020/07/ Informe-Integral- sobre-el-estado- ecol%C3%B3gico- del-Mar-Menor.pdf
J. Álvarez Rogel, C. García Alonso, F.J. Gilabert Cervera, R. Gómez Cerezo, V.M. León León, C. Marcos Diego, A. Pérez Ruzafa	Informe integral sobre el estado ecológico del Mar Menor. OCEANOGRAFÍA FÍSICA Y QUÍMICA	2017	Scientific report	https:// canalmarmenor.car m.es/wp-content/ uploads/2020/07/ Informe-Integral- sobre-el-estado- ecol%C3%B3gico- del-Mar-Menor.pdf
A. Faz Cano, J.B. Lobera Lössel, J. Mora Navarro,	Informe integral sobre el estado ecológico del Mar	2017	Scientific report	https:// canalmarmenor.car

P. Simón Andreu	Menor. DEPURACIÓN Y DESCONTAMINACIÓN DE LAS AGUAS			m.es/wp-content/uploads/2020/07/Informe-Integral-sobre-el-estado-ecol%C3%B3gico-del-Mar-Menor.pdf
Mediodes Consultancy on Environment and Landscape	Estudio preliminar de viabilidad de la construcción y uso de balsas de riego para retención temporal de salmueras y posterior reconversión de las balsas como filtro verde para la protección del Mar Menor.	2017	Report from a consultancy	Personal petition
A. Pérez Ruzafa	Informe de seguimiento del estado del Mar Menor	07/2017	Scientific monitoring report	https://canalmarmenor.carm.es/wp-content/uploads/2020/07/20170712_Informe-estado-Mar-Menor.pdf
A. Pérez Ruzafa	Informe de seguimiento del estado del Mar Menor	05/2018	Scientific monitoring report	https://canalmarmenor.carm.es/wp-content/uploads/2020/07/20180524_Informe-estado-Mar-Menor.pdf
P. García Moreno, A.D. Ibarra Marinas, J.M. Sánchez Balibrea (ANSE) y WWF España	La burbuja del regadío: el caso del Mar Menor. Evolución de los regadíos en el entorno del Mar Menor Campo de Cartagena 1977-2017	2018	Report from an environmental organization	https://www.asociacionanse.org/download/80/
A. Pérez Ruzafa	Informe de seguimiento del estado del Mar Menor	05/2019	Scientific monitoring report	https://canalmarmenor.carm.es/wp-content/uploads/2020/07/20190512_Informe-estado-Mar-Menor.pdf
Ministerio para la Transición Ecológica y el Reto Demográfico	Análisis de soluciones para el vertido cero al Mar Menor proveniente del Campo de Cartagena. Resumen ejecutivo después de la participación pública	2019	Public policy	https://www.chsegura.es/es/ciudadano/informacion-publica/analisis-de-soluciones-vertido-cero-al-mar-menor/
Grupo TRAGSA	Cuantificación, control de la calidad y seguimiento piezométrico de la descarga de agua subterránea del acuífero Cuaternario del Campo de Cartagena al Mar Menor. TTMM Varios. Murcia.	2020	Report from a consultancy	DOI: 07.831-0070/0411
J.M. Ruiz, M. Albentosa, B. Aldeguer, J. Álvarez-Rogel, J. Antón, M.D. Belando, J. Bernardeau,	Informe de evolución y estado actual del Mar Menor en relación al proceso de	2020	Scientific report	https://www.miteco.gob.es/es/prensa/informemarmenorju

J.A. Campillo, J.F. Domínguez, I. Ferrera, E. Fraile- Nuez, R. García, M. Gómez-Ballesteros, F.; Gómez, G. González-Barberá, F. Gómez-Jakobsen, V.M. León, C. López-Pascual, L. Marín-Guirao, C. Martínez-Gómez, J.M. Mercado, E. Nebot, A. Ramos, E. Rubio, J. Santos, F.; Santos, M. Vázquez-Luis, L. Yebra	eutrofización y sus causas. Informe de asesoramiento técnico del Instituto Español de Oceanografía (IEO). 165pp.			lio2020_tcm30-510566.pdf
J. Martínez Fernández, M.A. Esteve Selma (New Water Culture Foundation Observatory on Water Policies)	El colapso ecológico de la laguna del Mar Menor	2020	Report from an environmental organization	https://www.researchgate.net/publication/340965763_El_colapso_ecologico_de_la_laguna_del_Mar_Menor
Fundación Ingenio	Documento de posición técnica	2020	Report from an agricultural organization	https://fundacioningenio.com/wp-content/uploads/2021/12/DOCUMENTO-DE-POSICION-TECNICA-FUNDACION-INGENIO.pdf
A. Pérez Ruzafa	Informe de seguimiento del estado del Mar Menor	02/2020	Scientific monitoring report	https://canalmarmenor.carm.es/wp-content/uploads/2020/07/20200229_Informe-de-seguimiento-de-la-situaci%C3%B3n-del-Mar-Menor29022020.pdf
A. Pérez Ruzafa	Informe de seguimiento del estado del Mar Menor	05/2021	Scientific monitoring report	https://canalmarmenor.carm.es/wp-content/uploads/MAYO_Informe-de-seguimiento-de-la-situacio%CC%81n-del-Mar-Menor24mayo2021-1.pdf
A. Pérez Ruzafa	Informe de seguimiento del estado del Mar Menor	09/2021	Scientific monitoring report	https://canalmarmenor.carm.es/wp-content/uploads/Informe-de-seguimiento-de-la-situacio%CC%81n-del-Mar-

				Menor_09_septiembre_2021CIAMM.pdf
A. Pérez Pastor	Expert talk in the seminar ‘Hacia una agricultura medioambientalmente sostenible con el Mar Menor’	2021	Transcript from expert talk	https://www.youtube.com/watch?v=u2O6bnumBK4
J.L. García Aróstegui	Expert talk in the seminar ‘Hacia una agricultura medioambientalmente sostenible con el Mar Menor’	2021	Transcript from expert talk	https://www.youtube.com/watch?v=u2O6bnumBK4
J.T. García Bermejo	Expert talk in the seminar ‘Hacia una agricultura medioambientalmente sostenible con el Mar Menor’	2021	Transcript from expert talk	https://www.youtube.com/watch?v=u2O6bnumBK4
A. Aledo, J. Melgarejo	Expert talk in the seminar ‘Hacia una agricultura medioambientalmente sostenible con el Mar Menor’	2021	Transcript from expert talk	https://www.youtube.com/watch?v=u2O6bnumBK4
A. Aledo, G. Ortiz, J. Melgarejo	DIAGNÓSTICO SOCIAL ESTRATÉGICO DEL SECTOR AGRÍCOLA DEL CAMPO DE CARTAGENA	2021	Scientific report	https://www.crcc.es/wp-content/uploads/2018/06/INFORME-DIAGNOSTICO-SOCIAL-CRCC.pdf
Greenpeace	El Mar Menor, víctima del trasvase Tajo-Segura. Resumen	2021	Report from an environmental organization	https://es.greenpeace.org/es/sala-de-prensa/informes/mar-menor-victima-trasvase-resumen/
J.M. Ruiz, P. Clemente-Navarro, J.M. Mercado, E. Fraile-Nuez, M. Albentosa, L. Marín-Guirao, J. Santos	Nuevo evento de mortalidad masiva de organismos marinos en el Mar Menor: contexto y factores. Informe de asesoramiento técnico del Instituto Español de Oceanografía (IEO). 24pp.	2021	Scientific report	http://www.ieo.es/documents/10640/38594/informe+IEO_MarMenor_60921.pdf/36c4e2fc-7ab3-420d-b73d-8cf33478885e
Ecologistas en Acción	Propuesta de ordenación y franja de renaturalización en torno al Mar Menor	2021	Report from environmental organisation	https://www.ecologistasenaccion.org/wp-content/uploads/2021/09/propuesta_franja_renaturalizada.pdf
Gobierno de Murcia	Ley 3/2020, de 27 de julio, de recuperación y protección del Mar Menor.	2020	Public policy	https://www.boe.es/buscar/doc.php?id=BOE-A-2020-9793
Ministerio para la Transición Ecológica y el Reto Demográfico	Marco de Actuaciones Prioritarias para Recuperar el Mar Menor	2021	Public policy	https://www.miteco.gob.es/es/ministerio/planes-estrategias/mar-menor/marcodeactuacione

	sprioritariasspararec uperarelmarmenor_ 18022022_tcm30-536394.pdf

Table A2. Actors producing context-specific knowledge

Type of organization	Organization	Number of authors
Universities & Research Centers	Universidad de Murcia	26
	Universidad Politécnica de Cartagena	7
	Universidad de Alicante	6
	Universidad de Florida	2
	Earth and Environmental Sciences Division, Los Alamos National Laboratory	1
	École Polytechnique de Montréal	1
	Technical University of Catalonia	1
	Universidad Autónoma de Madrid	1
	Universidad Miguel Hernández de Elche	1
	CEBAS-CSIC	1
Governmental Research Organizations	Imida Instituto Murciano de Investigación y Desarrollo Agrario y Alimentario	1
	Instituto Español de Oceanografía	9
	Instituto Geológico y Minero Español	3
	Joint Research Center	1
Public & Private Consultancies	GRUPO TRAGSA	1
	FutureWater	2
	MEDIODES	1
Agricultural Organizations	Comunidad de Regantes Campo de Cartagena	1
	Fundación Ingenio	1
Environmental Organizations	Asociación de Naturalistas del Sureste (ANSE)	1
	Fundación Nueva Cultura del Agua	3
	Greenpeace	1
	WWF España	1
Public bodies	Comunidad Autónoma de la Región de Murcia	3
	D.G. Calidad y Evaluación Ambiental - CARM	2
	D.G. de Aguas de la Comunidad Autónoma de Murcia	2
	ESAMUR Entidad de Saneamiento y Depuración Región de Murcia	1
	Ministerio para la Transición Ecológica y el Reto Demográfico (MITERD)	4

Table A3. Twitter threads discussing controversies

Controversial theme	Twitter threads
What's the origin of the nutrients?	https://twitter.com/jotaortin/status/1451566952099483661<br>https://twitter.com/sosmarmenorofi/status/1451542687140696064<br>https://twitter.com/agronomopayes/status/1451528902107615234<br>https://twitter.com/agriCartagena/status/1500931520571322383<br>https://twitter.com/agronomopayes/status/1507019776916262919?t=urgY2_fPF2Udq3QXabq5qA&s=09<br>https://twitter.com/Defensa_Fuentes/status/1530243617075372032<br>https://twitter.com/Defensa_Fuentes/status/1496044599273529349<br>https://twitter.com/agroIngMarMenor/status/1510946506056122375<br>https://twitter.com/sosmarmenorofi/status/1539174375303397378<br>https://twitter.com/Mpenalver/status/1549776663554232320
How do nutrients travel to the lagoon?	https://twitter.com/aquifersudoe/status/1507406751267704838<br>https://twitter.com/GarciaArostegui/status/1509968616380379149?t=wR3LSzgPiHzz8YRcO6t2iw&s=08<br>https://twitter.com/Defensa_Fuentes/status/1513926579092701192<br>https://twitter.com/JosManu13437554/status/1518996669664305153<br>https://twitter.com/sosmarmenorofi/status/1542812464714227713<br>https://twitter.com/funingenio/status/1331925326595813376<br>https://twitter.com/edumeteo/status/1524723076214177792
Is current agriculture sustainable?	https://twitter.com/MarJungle/status/1442223567135928320?s=09<br>https://twitter.com/Fernandocvera/status/1442816690606297091<br>https://twitter.com/agriCartagena/status/1483804539199361024?t=eLYdbpjJ0wl5s_Ta60JeuA&s=09<br>https://twitter.com/MarianoSoto1972/status/1524303829738663937<br>https://twitter.com/agroIngMarMenor/status/1544793381993386000
How to avoid nutrient income?	https://twitter.com/RosaRodaNews/status/1502183304790949891<br>https://twitter.com/Defensa_Fuentes/status/1437340454631223299<br>https://twitter.com/VSinkevicius/status/1480856447504297993<br>https://twitter.com/EcoenAccionRM/status/1517237957962485761<br>https://twitter.com/MiguelCompany/status/1528650355604705282<br>https://twitter.com/MarianoSoto1972/status/1520300007949279232<br>https://twitter.com/RosaRodaNews/status/1531590100844429313<br>https://twitter.com/sosmarmenorofi/status/1542122703288782853<br>https://twitter.com/RosaRodaNews/status/1550367610805649409

Table A4. Uncertainty analysis on the causes of eutrophication (Cascade 1). Quotes included as examples

Cascade 1 - What is the origin of the nutrients?	Ambiguity	Incomplete knowledge	Unpredictability
Ecosystem		"Principales lagunas de conocimiento [...] El papel que están jugando los sedimentos en los ciclos de nutrientes en el Mar Menor se desconoce y tampoco hay referencias previas a la eutrofización generalizada que está teniendo lugar en estos momentos." Álvarez Rogel et al. 2017b<br><br>"La importancia del sedimento como fuente de N y P a la columna de agua se puso en evidencia en el proyecto BIOFOM" Ruiz et al. 2020.	"Los sucesos ocurridos recientemente en el Mar Menor y la enorme confusión sobre sus causas son en parte reflejo de un deficiente sistema de monitorización de la albufera, que debe ser mejorado y actualizado para detectar estos eventos y sus causas de forma inequívoca, fiable y transparente" Ruiz et al. 2021
Technology		.	"hacer estudios más profundos de gestión del alcantarillado en tiempo de lluvias para poder disminuir al máximo cualquier posibilidad de alivio" Faz Cano et al. 2017.
Society	"Los resultados obtenidos apuntan al origen mayoritariamente agrario de los nutrientes exportados desde la cuenca, como ocurre en otras cuencas agrícolas intensivas (Jordan et al. 1997; Kronvang 1999; Meissner etal. 2002; Lacroix et al. 2005)." Martínez Fernandez et al. 2013<br><br>"De este modo, aunque las entradas son de múltiples orígenes, ya que se detectan concentraciones relativamente altas tanto de nitratos como de fosfatos, con posible mezcla de aguas de	"Teniendo en cuenta que el contenido en P de las aguas subterráneas es muy bajo, los aportes de materiales terrígenos y de aguas residuales son las dos fuentes alóctonas principales de este elemento que, al estar asociado al material particulado, contribuyen a su depósito en el sedimento." Ruiz et al. 2020<br><br>"Algunas de las alegaciones indican que las balsas de almacenamiento de deyecciones ganaderas están impermeabilizadas y hay un cumplimiento de la normativa vigente, por lo que no consideran	" Principales lagunas de conocimiento [...] vertidos/descargas de aguas residuales de viviendas no conectadas a las redes de saneamiento o de las propias redes de saneamiento en mal estado." Alvarez Rogel et al. 2017b

	origen agrícola y urbano, están también forzadas por un nivel freático muy elevado, y como se muestra en este informe, las zonas de mayor influencia se desplazan en función de las actuaciones de gestión del agua.” Ruzafa, A. 2021.	oportuno la actuación de mejora de las instalaciones. Sin embargo, tras la visita de un equipo técnico a la zona, que un alto porcentaje de las instalaciones visitadas no cumplen las normas estipuladas de construcción, principalmente en materia de impermeabilización.” Ministerio para la Transición Ecológica y el Reto Demográfico 2019..

Table A5. Codebook

**THEMES**	**DESCRIPTION**
**THEMATIC NARRATIVES**	
**FRAMES**	
Frames Mar Menor	Refers to knowledge frames about the Mar Menor lagoon (what the problems and their drivers are)
Frames Campo de Cartagena	Refers to knowledge frames about the agricultural activity in Campo de Cartagena (what the problems and their drivers are)
**AGRICULTURE**	
Cattle and agricultural waste	Refers to descriptions of cattle production in Campo de Cartagena, the wastes its generates and their influence on lagoon's eutrophication
Crops	Refers to the type of crops cultivated in Campo de Cartagena, both past and present
Culture and territory	Refers to statements about the importance of agriculture for local culture and territorial development in Campo de Cartagena
Economic benefits	Refers to statements about economic benefits derived from the agricultural activity, their evolution and their distribution
Employment	Refers to statements about the employment generated by the agricultural sector in Campo de Cartagena, mostly connected with migrant workers
Erosion	Refers to descriptions of the problem of erosion in agricultural fields
España vaciada	Refers to claims about the demographic effect of the agricultural activity in Campo de Cartagena connected to migration patterns. The ‘España vaciada’ - Emptied Spain- concept is used to describe potential scenarios without agriculture in the area
Farm type	Refers to farming types in Campo de Cartagena

Fertilization	Refers to statements about farms fertilization practices in Campo de Cartagena
Illegal irrigation	Refers to claims and analysis of illegal irrigation in Campo de Cartagena
Inequality	Refers to claims about the unequal situation faced by different actors within the agricultural sector, especially migrant workers
Innovation SCR	Refers to claims about the innovation policies of agricultural companies, including both technological innovation and social corporate responsibility
Institutional support	Refers to statements about how public authorities have supported the expansion and intensification of agriculture in Campo de Cartagena
Markets competitiveness	Refers to the relations of Campo de Cartagena food industries with international food markets
Migrants	Refers to statements about migrants that came to Campo de Cartagena since the 90's to work in the fields
Multinationals	Refers to statements about multinational companies as relevant actors for the agricultural sector
Resilience	Refers to claims about resilience of the agricultural sector in Campo de Cartagena
Sustainability	Refers to claims about sustainability of the agricultural sector in Campo de Cartagena
Women in agriculture	Refers to mentions of the role of women in agricultural activities at Campo de Cartagena
**ECOLOGICAL STATUS**	
Caulerpa prolifera and *Cymodocea nodosa*	Refers to mentions to these two algae species, their role and dynamics within the lagoon ecology
Death and anoxia	Refers to the episodes of anoxia, hypoxia and euxinia in the lagoon as well as to the death of aquatic species triggered in such conditions
Health concerns	Refers to claims about the impacts on human health i related to the eutrophication of the Mar Menor lagoon
Lagoon ecology	Refers to descriptions of lagoon’s ecological dynamics
Lagoon status and eutrophication	Refers to statements about the ecological status of the lagoon, usually associated with certain indicators (concentration of nutrients, chlorophyll, salinity, oxygen, etc.) and to the eutrophication process and dynamics
Jellyfish	Refers to descriptions about jellyfish species in the lagoon, their ecological role and the problems they create to human activities like bathing or navigation
Nacra	Refers to claims about the status of *Pinna nobilis* (nacra) in the Mar Menor and their importance as emblematic species under threat of extinction
**WATER**	
Aquifer status	Refers to statements about the quantitative or qualitative status

	of the 5 aquifer layers in Campo de Cartagena
Storms	Refers to torrential rain events known as DANAs in the area which usually cause floods, erosion and vast inputs to the lagoon, ultimately driving anoxia in 2019
Floods	Refers to flood episodes and their impacts in urban areas and in the lagoon' eutrophication process
Desalination	Refers to desalination of seawater for irrigation
Desalobration	Refers to desalination of aquifer water for irrigation that produces brine full of nitrates for long dumped into the lagoon
Groundwater	Refers to groundwater dynamics in the watershed, including the 5 aquifer layers but mostly referring to the superficial one, the Quaternario
Groundwater - Runoff relations	Refers to interactions between surface and groundwater dynamics such as increment of aquifer table level contributing to permanent runoff
Inputs to lagoon	Refers to specific water-nutrient inflows to the lagoon from various sources in the watershed
Irrigation	Refers to water management and use for agricultural activities
Lagoon hydrodynamics	Refers to description of lagoon oceanography or water flow dynamics in relation to wind and internal currents
Surface water	Refers to surface hydrology and water flows
Tajo-Segura transfer	Refers to statements about the Tajo-Segura water transfer, its implications for agricultural productivity and intensification
Urban water	Refers to statements about (usually deficient) urban wastewater treatment and urban water management
Water sources	Refers to descriptions about water sources and uses in the watershed
Wetlands	Refers to descriptions of wetlands and their role as filters for agricultural water
**LAND**	
Biodiversity	Refers to analysis of biodiversity in the Mar Menor watershed
Ecosystem services	Refers to statements about the services provided by terrestrial ecosystems in the Mar Menor watershed
Land-Water relations	Refers to descriptions of land and water interrelations
Land use and soils	Refers to descriptions of land uses, land use change and types of soils in the watershed
Land use-biodiversity relations	Refers to descriptions of land use and biodiversity interrelations
Mines	Refers to descriptions about the unsealed mines and their impacts on the lagoon
Sediments	Refers to descriptions on the role of sediments as land-lagoon connectors, and in the lagoon's nutrient dynamics

NUTRIENTS

Phosphorous<br>Nitrogen	Refers to descriptions of nitrogen and phosphorus in their different forms, places and dynamics, mostly in relation to how they travel to the lagoon

RELATED PROBLEMS

Climate change	Refers to claims about the role of climate change in the Mar Menor eutrophication, often referred to future predictions or expectations
Other pressures	Refers to other pressures over the lagoon that are not directly associated to eutrophication, for instance harbors or beach regeneration
Social impacts	Refers to claims about social and economic impacts of the Mar Menor eutrophication
Fishing	Refers to mentions to the fishing sector and its affection by the Mar Menor eutrophication
Urban and tourism	Refers to statements about urban processes, like the rapid urbanization, and touristic activities
Lack of governance	Refers to claims about the lack of public action on the lagoon ecology and the absent role of decision makers
Responsibility	Refers to blame claims: who has the responsibility, who should act, who is not taking responsibility, etc.

SOLUTIONS

Close illegal irrigation	Refers to claims for 'closing the tap' to illegal irrigation areas
Agricultural governance and management	Refers to a wide range of measures related to the transition of the agricultural sector to make it compatible with the lagoon conservation, such as reduction of fertilizer use, of irrigated land or new markets for sustainable products
Agricultural precision	Refers to measures about improving the efficiency of fertirrigation through sensors and control of plant needs
Aquifer management	Refers to claims for reducing the level of water tables and managing the aquifer as an integral part of the basin
Close Tajo-Segura transfer	Refers to claims for closing the Tajo-Segura transfer
Coastal management	Refers to measures about coastal areas such as beaches or ports
Co-production	Refers to calls for collaboration among different actors, administrations or scientists in order to find better solutions, also to public participation in policy development
Denitrification	Refers to the process of nitrogen removal from nutrient-enriched water, usually groundwater or brine from desalination
Dilution	Refers to claims for opening the channels that connect the Mar Menor with the Mediterranean sea in order to increase water exchange and 'dilute' nutrient concentrations
Environmental governance	Refers to environmental policies and conservation measures
Environmental	Refers to environmental restoration of degraded sites that have

restoration	an impact on the lagoon
Floods and rrosion control	Refers to measures to control erosion and floods before they reach the lagoon
Monitoring	Refers to monitoring the implementation of measures in order to assess their effectiveness
More knowledge	Refers to new knowledge that is needed to propose effective solutions
Other sectors	Refers to measures to support economic sectors to transform their economy and practices while reducing their impacts, including fishing, navigation and cattle raising
Policies and institutions	Refers to claims about how existing or newly created policies and governance structures can be used to enact solutions
Restoration	Refers to measures that seek the restoration of water quality and ecological dynamics within the lagoon, such as using planting oysters to filter nutrients from water
Soil conservation	Refers to measures that improve the quality of soils in the basin in order to prevent erosion and nutrient leakage
Soil decontamination and mine restoration	Refers to the decontamination of polluted soils and the restoration of mines to prevent heavy metal leackage
Gray solutions	Refers to infrastructural solutions such as pumping surface runoff to a denitrification plant and reuse it for irrigation
Nature-based solutions	Refers to solutions involving ecological processes such as the use of wetlands as natural filters for nutrients and floods retention
Control of sources (Zero Discharge)	Refers to claims for reducing all sort of inputs to the lagoon, including water and nutrients inputs
Urban governance	Refers to measure over urban areas, urbanism and touristic activities
Wastewater treatment	Refers to claims for improving wastewater treatment systems because they collapse during DANAs
Water governance and management	Refers to water governance and management measures
**DIALOGIC NARRATIVES**	
**WHO**	
Author	Codes names of authors of the different reports analyzed
Discipline	Codes the disciplines of the authors as stated in the reports
Institution	Codes authors’ institutional adscriptions
**WHAT**	
Knowledge gap	Refers to gaps identified in the scientific literature that the paper aims to address
Research goal	Refers to research goals in scientific papers and reports
**WHY**	

Governance challenge	Refers to management or governance challenges used by the author as justifications for their work
Normative goals	Refers to values or positionality of authors within the socioenvironmental conflict, especially those from environmental and agricultural organizations
Problem framing	Refers to general scientific/environmental problem addressed by the study (not specifically to how the Mar Menor or Campo de Cartagena are framed)
**HOW**	
Conceptual framing	Refers to conceptual frameworks or relevant concepts from the literature that are used to frame the paper. For instance resilience, ecosystem services, adaptive governance, etc.
Data	Refers to data collected and used for the analysis
Methods	Refers to explicit mentions and descriptions of methods used for data gathering and analysis
**RELATIONS**	
Alignment/Addition	Refers to citations upon which the author builds or supports
Contestation	Refers to explicit contestations of other authors' arguments or results. Related to Ambiguities
Collaboration	Refers to explicit mentions about collaborations between different authors or between authors and other actors in the Mar Menor
Communication	Refers to calls for improving public communication of scientific knowledge or particular actors views
**UNCERTAINTIES**	
Ambiguity	Refers to disagreements or different views on particular issues
Incomplete Knowledge	Refers to claims about limited knowledge on particular issues that can be address through more research and knowledge, includes claims for more research needed
Data and methods	Subtheme in Incomplete Knowledge. Refers to declared uncertainties in data and methods
Assumption	Subtheme in Incomplete Knowledge. Refers to assumptions made by authors in their methods or in their results
Unpredictability	Refers to claims about limited knowledge on particular issues that cannot be addressed through more research and knowledge at the moment
Management of uncertainty	Refers to explicit or implicit strategies to handle uncertainties
**KNOWLEDGE**	
Certainties	Refers to statements about the analyzed problem that are considered truths or at least certainties with a high degree of confidence in current knowledge
Complexity	Refers to statements about the complexity of the Mar Menor problem and the difficulty of addressing it from a single discipline

Evidence based policy	Refers to calls for evidence based policy, more about desires than about actual effective implementation of this model
Hyper known object	Refers to statements about the high degree of knowledge accumulated on the Mar Menor lagoon over the past few decades
